# Prediction of fluid responsiveness. What’s new?

**DOI:** 10.1186/s13613-022-01022-8

**Published:** 2022-05-28

**Authors:** Xavier Monnet, Rui Shi, Jean-Louis Teboul

**Affiliations:** AP-HP, Service de médecine intensive-réanimation, Hôpital de Bicêtre, DMU 4 CORREVE, Inserm UMR S_999, FHU SEPSIS, CARMAS, Université Paris-Saclay, 78 rue du Général Leclerc, 94270 Le Kremlin-Bicêtre, France

**Keywords:** Passive leg raising, Tidal volume, Fluid challenge, Volume expansion, Cardiac output, Fluid balance

## Abstract

Although the administration of fluid is the first treatment considered in almost all cases of circulatory failure, this therapeutic option poses two essential problems: the increase in cardiac output induced by a bolus of fluid is inconstant, and the deleterious effects of fluid overload are now clearly demonstrated. This is why many tests and indices have been developed to detect preload dependence and predict fluid responsiveness. In this review, we take stock of the data published in the field over the past three years. Regarding the passive leg raising test, we detail the different stroke volume surrogates that have recently been described to measure its effects using minimally invasive and easily accessible methods. We review the limits of the test, especially in patients with intra-abdominal hypertension. Regarding the end-expiratory occlusion test, we also present recent investigations that have sought to measure its effects without an invasive measurement of cardiac output. Although the limits of interpretation of the respiratory variation of pulse pressure and of the diameter of the vena cava during mechanical ventilation are now well known, several recent studies have shown how changes in pulse pressure variation itself during other tests reflect simultaneous changes in cardiac output, allowing these tests to be carried out without its direct measurement. This is particularly the case during the tidal volume challenge, a relatively recent test whose reliability is increasingly well established. The mini-fluid challenge has the advantage of being easy to perform, but it requires direct measurement of cardiac output, like the classic fluid challenge. Initially described with echocardiography, recent studies have investigated other means of judging its effects. We highlight the problem of their precision, which is necessary to evidence small changes in cardiac output. Finally, we point out other tests that have appeared more recently, such as the Trendelenburg manoeuvre, a potentially interesting alternative for patients in the prone position.

## Background

Fluids, administered to patients with shock and hypotension in the operating room or intensive care unit (ICU), are drugs. On the one hand, their effect is inconstant, due to inter-individual variability in the relationship between cardiac output and preload [1]. When fluid responsiveness is not assessed, a fluid bolus increases cardiac output in only half of the cases. This means that many patients will inadvertently receive fluids while there is no cardiac output response [1, 2]. On the other hand, the deleterious effects of fluid administration are now clearly demonstrated. Increase in fluid balance is a factor independently associated with mortality of patients in shock, especially during septic shock [3], and with an increased rate of complications after surgery [4].

Therefore, it is logical to condition the infusion of fluid boluses on the prediction of their effectiveness. The main goal of this prediction is to avoid administering ineffective fluid, which would only have deleterious effects without generating any benefit. For this purpose, several tests and indices have been developed.

The aim of this review is to provide an up-to-date summary of knowledge regarding these tests and indices, emphasizing the literature published in the last three years. What do recent studies add regarding their reliability? How should they be performed in practice, and how should their effects be assessed? What is new regarding their conditions of use? What new tests have been developed? These are the questions we will try to answer.

## Passive leg raising

### What do we already know about the test?

Transferring a patient from a semi-recumbent position to a position where the trunk is horizontal and the lower limbs are elevated at 30–45° mobilizes blood from the splanchnic territory and the lower limbs and significantly increases mean systemic pressure, the upstream pressure of systemic venous return [5]. The passive leg raising (PLR) test increases cardiac preload and allows the assessment of preload responsiveness of both ventricles. The advantage of this “self-transfusion” of roughly 300 mL of blood [6] is that it is reversible. In addition, the test does not depend on ventilation and heart rate, and it remains reliable in patients with spontaneous ventilation and cardiac arrhythmia [7].

The reliability of the PLR test for detecting preload responsiveness is well established, after the publication of numerous studies [8] and some meta-analyses [9, 10]. The test not only has good sensitivity and specificity (85 and 91%, respectively) [9], but also very good positive and negative predictive values and likelihood ratios [11]. Use of the test has grown [7] and it is recommended in the haemodynamic management of septic shock by the Surviving Sepsis Campaign [12].

However, it has long been clear that unfortunately the haemodynamic effects of PLR cannot be measured on simple arterial pressure measured invasively [9] and, a fortiori, non-invasively [13]. Even changes in arterial pulse pressure, which is best related to stroke volume, only imperfectly follow the effects of PLR on cardiac output, as is the case for the fluid challenge [14]. Thus, the effects of PLR should be measured directly on cardiac output (Table 1).

**Table 1:** Characteristics of tests assessing preload responsiveness by mimicking a classic fluid challenge

Test	Advantages	Limitations	Confounding factors	Criterion of judgement	Diagnostic threshold	Level of evidence*
Passive leg raising	→Reversible, no fluid infusion	→Requires a direct estimation of CO/SV	→Possible false negatives in case of intra-abdominal hypertension →False negatives in case of venous compression stockings	↗ CO	≥ 10%	++++
	→Works regardless of breathing activity, cardiac rhythm, Vt, lung compliance	→False negatives in case of IAH		↗ VTI	≥ 10%	++++
	→Very well validated			↗ end-tidal CO_2_	≥ 5% ≥ 2 mmHg	++
				↗ perfusion index	≥ 9%	+
				↘ PPV/SVV	≥ − 1 to 4 points	+
				↘ capillary refill time	≥ − 27%	+
Mini-fluid challenge	→Easy to perform	→Requires a direct estimation of CO/SV	→Poor precision of the technique measuring cardiac output →Volume of fluid infused (minimum: 100 mL)	↗ CO	≥ 5%	++
	→Works regardless of breathing activity, cardiac rhythm, Vt, lung compliance, IAP	→Requires a precise estimation of CO/SV →Still requires fluid infusion		↗ VTI	≥ 10%	+
Trendelenburg manoeuvre	→Reversible, no fluid infusion →Possible even in prone position, on the operating table or under ECMO →Works regardless of breathing activity, cardiac rhythm, Vt, lung compliance	→Possible gastric reflux →Requires more validation	→Intra-abdominal hypertension?	↗ CO	≥ 8 to 10%	+

*CO* cardiac output, *IAH* intra-abdominal hypertension, *ECMO* extracorporeal membrane oxygenation, *PPV* pulse pressure variation, *SV* stroke volume, *SVV* stroke volume variation, *Vt* tidal volume under mechanical ventilation, *VTI* velocity-time integral in the left ventricular outflow tract

^*^Takes into account the number of positive studies (confirming reliability) and of negative studies (denying reliability)

**Fig. 1:**
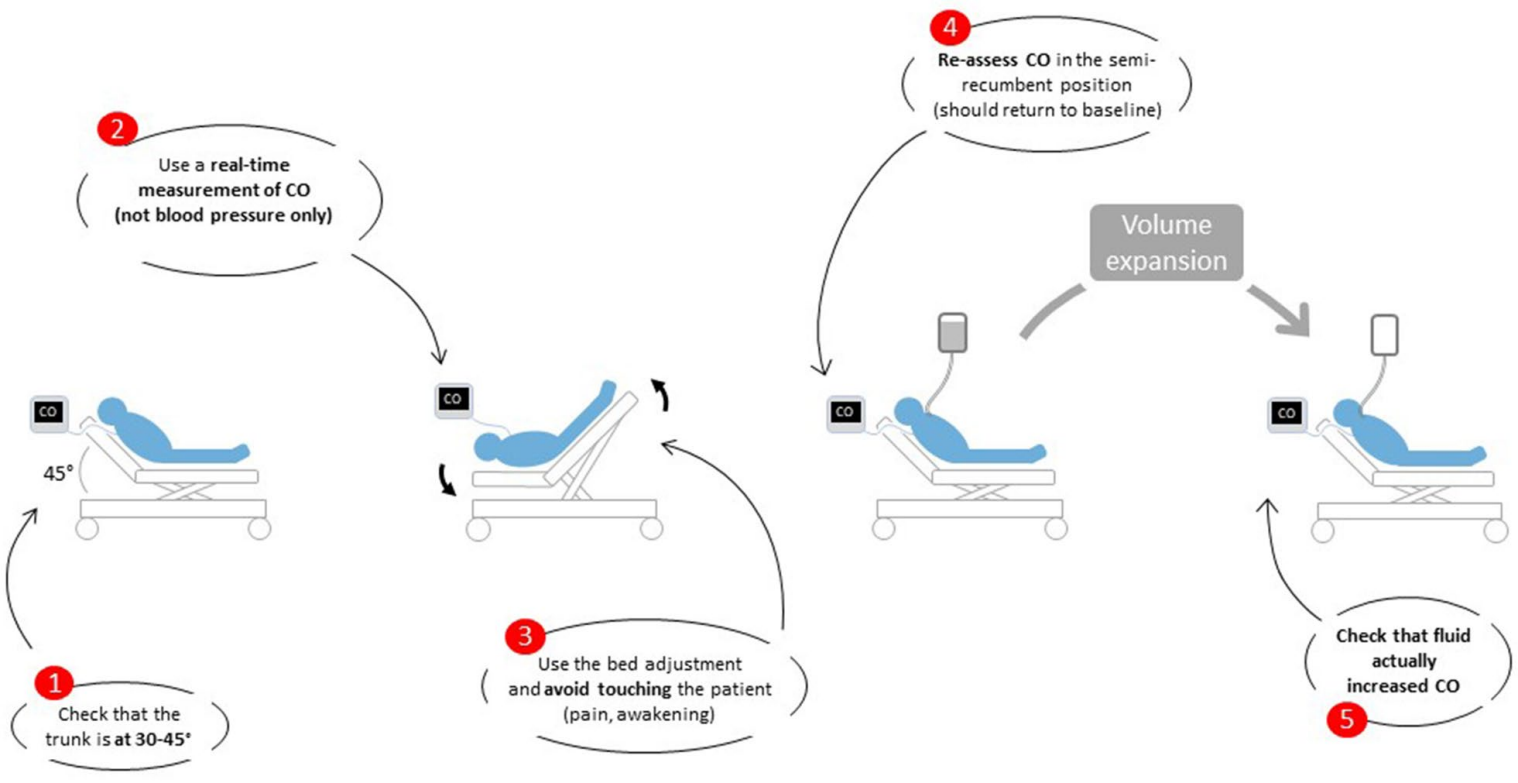
Practical rules for performing passive leg raising. *CO* cardiac output

What haemodynamic monitoring techniques can be used for this purpose? Since the effects of PLR are sometimes limited in time (the increase in cardiac output peaks in the minute after starting the test, and then diminishes in some patients), cardiac output must be estimated in real time (Fig. 1). Pulse wave analysis is perfectly suited and simple to use [15]. Even though the estimation of cardiac output by uncalibrated systems is less reliable when arterial tone changes, which makes these techniques unsuitable for intensive care patients [16], they remain reliable for estimating the effects of PLR since it does not modify vascular resistance. This is also true when PLR is assessed with the volume clamp technique, which estimates cardiac output through the uncalibrated analysis of an arterial curve obtained non-invasively through a finger cuff [17]. Echocardiography and oesophageal Doppler, which estimate stroke volume beat-to-beat, are quite suitable. With cardiac ultrasound, for the sake of the precision of the measurement, it is wise to keep the probe on the patient’s skin and the ultrasound beam in the left ventricular outflow tract (LVOT). In ventilated patients in the absence of any spontaneous respiratory activity, changes in end-tidal carbon dioxide measured at the tip of the tracheal tube reflect changes in cardiac output and allow monitoring of the effects of the PLR test [18–20]. This offers an attractive alternative to previous methods, especially during surgery and anaesthesia.

### What's new?

#### Monitoring technique

In recent years, many more studies have been devoted to the description of alternative methods to estimate the haemodynamic effects of PLR, less invasive than pulse contour analysis and simpler to perform than echocardiography.

This is the case for bioreactance (Starling, Baxter, Deerfield, USA), at least in its latest version, in which the duration over which cardiac output values are averaged and the frequency at which their refreshed display are compatible with the duration of the PLR effects [21]. However, the repeatability of bioreactance cardiac output measurements during PLR has recently been questioned [22]. Bioimpedance systems, which are more subject to artifacts than bioreactance systems, seem to be less reliable [23].

Another non-invasive and easy-to-use solution may come from the plethysmography signal. Its amplitude is estimated by the perfusion index, the ratio between its pulsatile portion, which is displayed on bedside screens, and the non-pulsatile portion. This index is determined by vasomotor tone, which decreases its amplitude, and stroke volume, which increases it [24]. Then, even though it does not provide an absolute value of cardiac output, it may follow its trends. The respiratory variation of the perfusion index, quantified by the pleth variability index, has been demonstrated to indicate preload responsiveness [25], even though some poorer results have been reported in critically ill patients receiving norepinephrine [26], which may alter the quality of the plethysmographic signal.

During PLR, two studies by our group showed that the increase in perfusion index, which is automatically measured by some monitors, followed changes in cardiac output during a PLR test and that these changes detected a preload responsive state [27, 28]. An unstable signal in some patients could be a limitation of the method [28]. These results should be confirmed by further studies by other groups, but suggest that plethysmography might be a non-invasive, cheap, and widely used alternative to all previous techniques used to estimate PLR effects.

Another attractive way to assess PLR effects might be to assess changes not in a surrogate of cardiac output but in the respiratory variation of arterial pulse pressure (PPV) or stroke volume (SVV) in mechanically ventilated patients. Indeed, it has recently been reported that the decrease in PPV induced by a PLR test, reflecting the decrease in preload responsiveness induced by the preload challenge, detects preload responsiveness [29, 30] (Table 1). Likewise, another study, conducted on patients with protective ventilation in a cardiac surgery ICU, reported similar results, this time with a decrease in SVV [31] (Table 1). However, the best diagnostic threshold is not well defined at the moment, as studies are few and provide different values (Table 1). In addition, these relatively low diagnostic thresholds of PPV drops may pose the problem of measurement accuracy.

Preload responsiveness could even be detected by the effects of the PLR test on capillary refill time [32], although it reflects skin perfusion and sympathetic vasoconstriction without being a direct surrogate of cardiac output. A decrease of 27% or more was the reported diagnostic threshold (Table 1). However, this threshold was close to the reproducibility limit of the measurement. Moreover, it was performed in a very standardized way (standardized pressure applied on the skin, video recording of the skin colour under standard lighting, etc.) [32]. Thus, the method might be difficult to implement in practice. Moreover, other studies should confirm that there is a close relationship between capillary refill time and cardiac output, as they are not directly related physiologically.

The use of carotid or femoral arterial flow during the PLR test is more doubtful. Indeed, published studies are highly contradictory, some showing that these arterial flows follow the changes induced by the PLR test [33], while others demonstrate the method is unreliable [34, 35]. More generally, doubts exist regarding the ability of carotid blood flow to track changes in cardiac output [36, 37]. One should thus be cautious before considering this technique for clinical practice.

#### Limitations

PLR testing is contraindicated in patients with intracranial hypertension, and it is difficult to perform during surgery. Venous compression stockings likely result in false negatives by reducing the mobilized blood volume [38]. However, the test is still valid in circumstances that limit the use of PPV and SVV, such as spontaneous breathing, cardiac arrhythmia, low tidal volume (Vt) ventilation, low lung compliance, or right heart failure [38].

More recently, a study suggested that intra-abdominal hypertension, which would reduce the splanchnic blood volume mobilized during PLR, or even interrupt the inferior vena cava flow by a waterfall phenomenon, would generate some false negatives [39]. The results of this single study need to be confirmed [40] but, in the meantime, it is reasonable to advise caution in interpreting the PLR test in cases of intra-abdominal hypertension (Table 1).

## End-expiratory occlusion test

### What do we already know about the test?

As PPV and SVV, the end-expiratory occlusion (EEO) test detects preload dependence by taking advantage of cardiopulmonary interactions [41]. In mechanically ventilated patients, each insufflation increases intrathoracic pressure, thereby increasing right atrial pressure and decreasing right cardiac preload. Thus, interrupting ventilation in expiration for a few seconds, while the alveolar pressure is maintained at the level of positive end-expiratory pressure (PEEP), stops this cyclical decrease in cardiac preload. In preload-responsive patients, cardiac output significantly increases [42]. Studies testing the reliability of the EEO test have been the subject of three recent meta-analyses [43–45] confirming its reliability. The diagnostic threshold is a 5% increase in cardiac output [43] (Table 2).

**Table 2:** Characteristics of tests and indices assessing preload responsiveness based on heart–lung interactions

Test/index	Advantages	Limitations	Confounding factors	Criterion of judgement	Diagnostic threshold	Level of evidence
PPV	→Automatically measured →Widely available (invasive or non-invasive arterial pressure curve) →Requires no manoeuvre →Very well validated	→Impossible to use in many patients because of confounding factors	→False positives in case of cardiac arrhythmias, spontaneous breathing activity and possibly right ventricular failure →False negatives in case of low Vt, low lung compliance and IAH	Absolute value itself	≥ 15%	++++
SVV	→Automatically measured →Requires no manoeuvre →Well validated	→Impossible to use in many patients because of confounding factors →Requires a device for pulse contour analysis	→Those of PPV →An arterial pressure of poor quality may provide wrong values	Absolute value itself	≥ 15%	+++
EEO test	→Easy to perform →Works regardless of breathing activity, cardiac rhythm, Vt, lung compliance →Well validated	→Requires a direct estimation of CO/SV →Requires mechanical ventilation →Cannot be used if the patient interrupts the 15-s EEO	→Interruption of the test before its end by breathing efforts of the patient	↗ CO	≥ 5%	+++
				↗ VTI (better with additional EIO)	EEO alone: ≥ 5% EEO + EIO: ≥ 13%	+
				↗ perfusion index	≥ 2.5%	+
Vt challenge	→Requires no measurement in CO/SV (just an invasive or non-invasive arterial pressure curve) →Reliable in prone position and in spontaneously breathing patients	→Requires mechanical ventilation →Different diagnostic thresholds reported →Requires more validation	→Cardiac arrhythmias? →Intra-abdominal hypertension?	↗ PPV	≥ 1 to 3.5%	++
Vena cava distensibility	→Requires no measurement in CO/SV	→False positives in case of spontaneous breathing activity and possibly right ventricular failure →False negatives in case of low Vt, low lung compliance →Quite low reliability →Not reliable in case of IAH →For SVC: requires TOE	→Those of PPV (except cardiac arrhythmia)	Absolute value itself	IVC: ≥ 12% SVC: ≥ 12 to 36%	+

*CO* cardiac output, *EEO* end-expiratory occlusion, *EIO* end-inspiratory occlusion, *IAH* intra-abdominal hypertension, *IVC* inferior vena cava, *PPV* pulse pressure variation, *SV* stroke volume, *SVC* superior vena cava, *SVV* stroke volume variation, *TOE* trans-oesophageal echocardiography, *Vt* tidal volume under mechanical ventilation, *VTI* velocity-time integral in the left ventricular outflow tract

^*^Takes into account the number of positive studies (confirming reliability) and of negative studies (denying reliability)

The duration of the EEO must be longer than 12 s [42], to allow the increased preload to be transmitted from the right to the left cardiac side (pulmonary transit time) and also to allow devices that average cardiac output values over several seconds to display this increase. Thus, the test is not feasible in patients interrupting the end-expiratory pause because of too-marked respiratory activity [42] (Table 2).

**Fig. 2:**
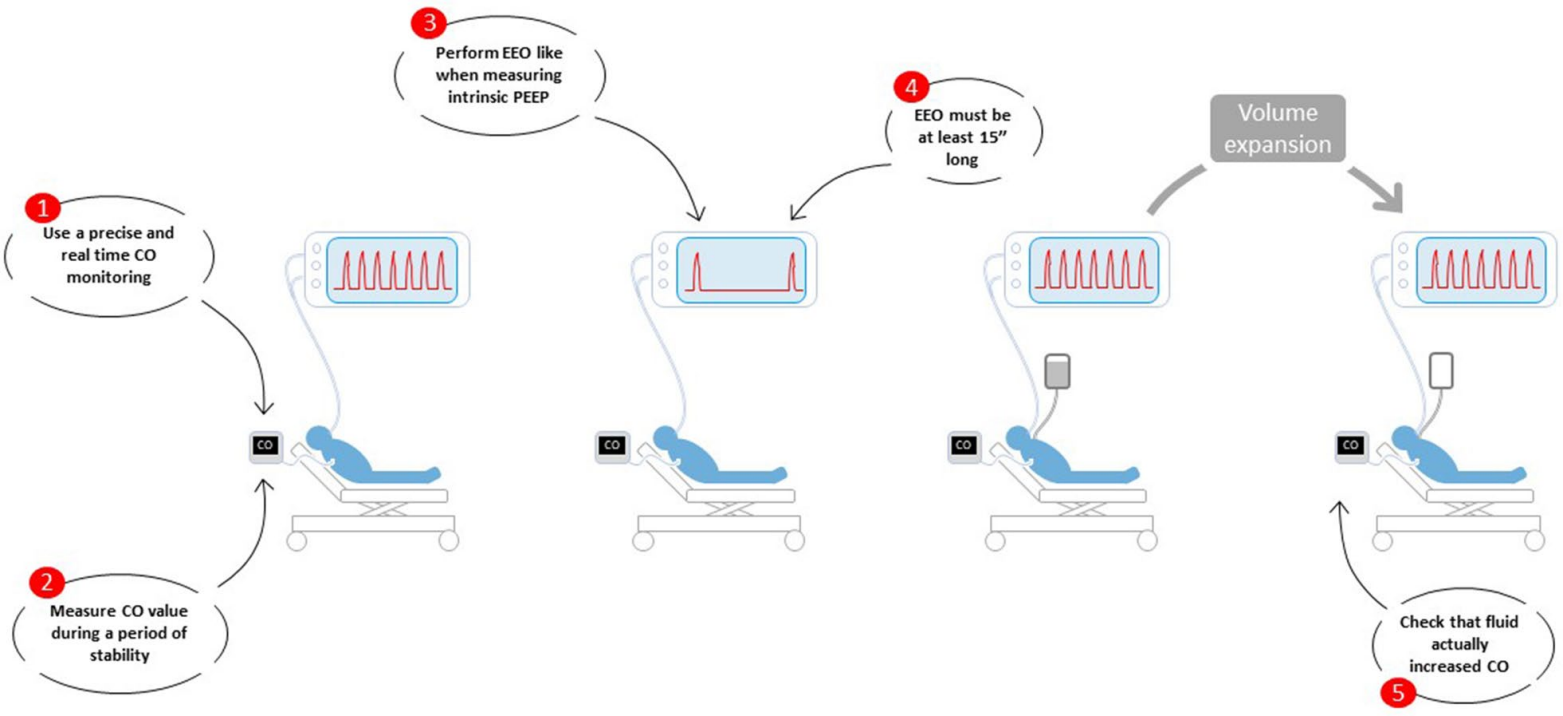
Practical rules for performing an end-expiratory occlusion test. *CO* cardiac output, *EEO* end-expiratory occlusion test, *PEEP* positive end-expiratory pressure

On the other hand, the advantage of the test is that it is easy to perform (Fig. 2). It is valid even in the event of small Vt or cardiac arrhythmia and in patients with spontaneous respiratory activity, provided that it is not too marked [42] (Table 2).

The effects of the EEO test are fleeting, as they begin to wear off as soon as mechanical ventilation resumes. In addition, they are relatively small, so, therefore, is the diagnostic threshold. Thus, measuring the effects of the EEO test requires an estimate of cardiac output that is both in real time and precise [42] (Fig. 2).

For this purpose, the drawback of arterial pulse pressure is that its changes during a 15-s EEO cannot be seen simply on bedside monitors [46]. The pulse wave contour analysis, a real-time and very precise measurement (the smallest detectable significant change is of the order of only 1–2% [47]) is perfectly adapted but is an expensive and invasive technique.

### What's new?

#### Monitoring technique

As with the PLR test, several recent studies have looked at the method that can be used to detect the effects of the EEO test. The drawback of cardiac ultrasound for measuring the effects of the test is that its precision is relatively low. As underlined before, the smallest detectable change in stroke volume (i.e., changes in the velocity-time integral (VTI) in the LVOT) is 10%, below the diagnostic threshold for the EEO test [48]. Therefore, it was suggested to combine a 15-s EEO with a 15-s end-inspiratory hold, separated from the EEO by a resumption of mechanical ventilation [49]. During the end-inspiratory occlusion, conversely to what occurs during EEO, stroke volume increases more in the case of preload responsiveness than of preload unresponsiveness. The addition of the VTI changes (in absolute value) observed during the two respiratory holds allows one to detect preload responsiveness with good diagnostic reliability and with a threshold of 13% [48] compatible with the precision of transthoracic echocardiography [49]. This combination of end-inspiratory and EEO tests, with repeated measurements of the VTI in the LVOT, takes time. However, it opens up the possibility of assessment by echocardiography, which is often the only cardiac output measurement technique available.

When using oesophageal Doppler, which has the same precision issues as cardiac ultrasound, the combination of EEO and end-inspiratory occlusion can also be used [50]. It was recently shown that a portable carotid Doppler tool could also track changes in stroke volume during combined end-inspiratory and end-expiratory occlusions [51], a result that is interesting but needs confirmation.

In one study, changes in the plethysmographic perfusion index were shown to closely follow changes in the cardiac index during EEO, as it did during PLR [27]. A diagnostic threshold of 2.5% was obtained (Table 2), while the smallest significant change in the perfusion index was 2% [27]. As for PLR, although this single study opens up the prospect of easy monitoring of the EEO test, it must be confirmed.

Finally, tools that track changes in cardiac output or stroke volume during the EEO test must be able to capture relatively brief effects. Thus, the commercial version of the bioreactance system, which averages cardiac output over 24 s, cannot track the effects of the test. However, a modified version bioreactance system that reduces the cardiac output averaging time to 8 s was recently shown to be quite appropriate [27]. Such results highlight the fact that the technical characteristics of monitoring tools must be carefully considered.

#### Limitations

One study reported that the EEO test was reliable at 8 mL/kg Vt but not at a 6 mL/kg Vt [52]. However, several studies which clearly demonstrated the validity of the EEO test had included ventilated patients with a Vt less than 8 mL/kg, and even less than 7 mL/kg [42, 53]. This was even recently confirmed by a meta-analysis that only included studies performed under conditions of low Vt [54]. In addition, the EEO test has been shown to be reliable regardless of the level of respiratory driving pressure [55]. Thus, low Vt ventilation is likely not a limitation of the EEO test.

Two studies also suggested that the EEO test was unreliable in the prone position [56, 57]. In one of the studies, reliability was only acceptable in patients whose central venous pressure increased during EEO [56]. However, there is no obvious reason why the test should be less reliable in the prone position than in the supine position, and this point also needs confirmation.

Finally, a study recently suggested that the EEO test loses sensitivity during laparoscopic surgery [58]. It is possible that the decrease in transdiaphragmatic pressure explains this possible limitation of the test, but this requires confirmation.

## Pulse pressure and stroke volume variations

### What do we already know about the indices?

PPV, which is an easy way to quantify the decrease in systolic arterial pressure induced by the inspiratory decrease in cardiac preload under mechanical ventilation (delta down), is the preload responsiveness index that has the highest level of evidence [59]. However, PPV and SVV are limited by the fact that they cannot be used in many clinical conditions, which can be easily remembered through the acronym LIMITS [60]: Low heart rate/respiratory rate ratio (extremely high respiratory rates), which creates some false negatives, Irregular heartbeats (false positives), Mechanical ventilation with low tidal volume (false negatives), Increased abdominal pressure (false positives), Thorax open (false negatives) and Spontaneous breathing (false positives) (Table 2). Then, PPV and SVV can only be used in a minority of patients, especially in the ICU [59].

### What's new?

#### Limitations

The question of false positives of PPV induced by right heart failure remains unresolved. The reality of this theoretical limitation is not certain, as it was evaluated by only one human study with many limitations [61]. A recent study in pigs suggests that elevation in the level of PEEP and the accompanying increase in right ventricular afterload increase PPV independently of variations in preload [62]. However, the animals in this study did not have right ventricular failure [62]. Despite these limitations, recent studies have shown that the relative changes in PPV and SVV may help assess fluid responsiveness, even in cases where its absolute value is not interpretable. During tests that are usually performed by assessing changes in cardiac output, changes in PPV and SVV might be used as surrogates of cardiac output, allowing one to perform these tests with a simple arterial line and no haemodynamic monitor. Changes in PPV and/or SVV may allow one to assess the PLR test [29–31] as mentioned above and the mini-fluid challenge as detailed below [63, 64].

## Tidal volume challenge

### What do we already know about the test?

This test makes it possible to overcome the unreliability of PPV in the event of low Vt ventilation. When Vt is at 6 mL/kg of predicted body weight, the test consists of increasing it transiently from 6 to 8 mL/kg and measuring the induced changes in PPV [52]. If the absolute value of PPV increases significantly, this implies that both ventricles are preload-dependent (Table 2).

The Vt challenge has been validated by far fewer studies than the PLR test, for example, although its reliability has been confirmed in a recent meta-analysis [54]. Moreover, the diagnostic thresholds differ slightly between the few available studies (Table 2), which in addition expressed the change in PPV either as an absolute value [30, 52, 65]} or as a percent change [56, 66, 67], or both [29].

### What's new?

Recent publications suggest the interest in the Vt challenge in the operating room context [66, 67], where the PLR test is not feasible during many interventions. In particular, the test has been demonstrated to be reliable in patients in prone position during neurosurgery [57]. If confirmed in patients with ARDS in the ICU, this would provide a solution in this context, where PLR cannot be used.

The test can be used in patients with some spontaneous breathing under mechanical ventilation [30]. This has been demonstrated with PPV. However, when the effects of the Vt challenge are assessed on the changes not in PPV but in inferior vena cava distensibility, the diagnostic value of the test seems to be notoriously insufficient as shown by another recent study [29]. This is probably due to the lower intrinsic reliability of the inferior vena cava variations, as detailed below.

## Respiratory variation in the diameter of the vena cava

### What do we already know about the index?

Variation in the diameter of the vena cava has long been proposed for the detection of preload dependence in mechanically ventilated patients [68]. The advantage of this index is that it can be easily obtained using transthoracic cardiac ultrasound, without requiring extensive experience in ultrasound. Conversely, the collapsibility of the superior vena cava requires transoesophageal ultrasound, which requires more experience [41] (Table 2).

The distensibility of the vena cava unfortunately shares with PPV and SVV the fact that it cannot be used when Vt is low, as has been recently shown [29], when lung compliance is low and when there is spontaneous breathing (Table 2). Intra-abdominal hypertension considerably impairs the reliability of this index. In addition, as detailed below, recent publications have confirmed the low reliability of these indices of fluid responsiveness.

### What's new?

#### Reliability

The reliability of variations in the diameter of the vena cava has been reported to be quite low in several studies and meta-analyses published in recent years [69–71]. A large study confirmed that changes in diameter, especially of the inferior vena cava, have low diagnostic sensitivity and specificity in detecting preload responsiveness, as assessed by the PLR test [72]. Only the extreme values, observed in very few patients, were informative [72].

This may have a physiological explanation. Variations of the vena cava do not depend only on the state of preload responsiveness, but on other factors as well. For the inferior vena cava, while it is true that its intramural pressure, the central venous pressure, varies more in amplitude in the event of preload responsiveness [73], which tends to vary its diameter, it is not the only factor that comes into play. Compliance of the vena cava, which is supposed to be higher in the case of hypovolemia, extramural pressure, i.e., the intra-abdominal pressure, and its respiratory variations, which depend on the thoracoabdominal transmission of the intrathoracic pressure, also play a role. Accordingly, it has been shown that the diagnostic value of variations in the inferior vena cava becomes very low during intra-abdominal hypertension [72]. Moreover, the site of measurement of inferior vena cava diameter affects the accuracy of its variability for predicting fluid responsiveness [74–76].

However, in septic non-intubated patients, a recent study suggested that preload responsiveness was detected by changes in the diameter of the inferior vena cava induced by a standardized respiratory manoeuvre [77]. Nevertheless, the echographic measurement should be performed 4 cm from the right atrium [74], which might be challenging, especially in the semi-recumbent position during respiratory distress [76]. In addition, patients must be able to cooperate and standardize their breath, and must have no active expiration [76].

In addition to intra-abdominal hypertension, vena cava distensibility shares with PPV some conditions in which it is less reliable: spontaneous breathing, low tidal volume and lung compliance (Table 2). Considering all these limitations, it is surprising to see that vena cava distensibility is often thought to be the key index for assessing fluid requirements in point-of-care ultrasound [78]. Its ease of measurement, and the few skills that are necessary for assessing it, do not compensate for its lack of reliability. With echocardiography, preload responsiveness is better assessed with the PLR test, the EEO test combined with expiratory tests or with the mini-fluid challenge.

## Mini-fluid challeng﻿e

### What do we already know about the test?

The most obvious way to detect preload responsiveness is to infuse a bolus of fluid and measure its effects on cardiac output. The fluid challenge has for years been proposed to guide fluid therapy [79].

However, it is easy to understand that a "classic" fluid challenge, with the infusion of 200–500 mL of fluid, is not a "challenge", but the treatment itself. If there is no preload responsiveness, which occurs in half of the cases, it is not possible to withdraw the fluid administered in excess. The classic fluid challenge inevitably leads to fluid overload. In addition, the fact that cardiac output increases following the infusion of 200–500 mL of fluid does not necessarily imply that this will be the case for the next bolus. The volume is indeed large enough to convert preload-dependent ventricles into independent ones.

Therefore, the idea of administering only a “mini-fluid challenge”, performed with a quite small volume of fluid to assess preload responsiveness is interesting [80]. It consists in infusing 100 to 150 mL of crystalloid or colloid over 60 to 120 s and measuring the response of cardiac output or one of its surrogates. This response predicts the effects on cardiac output of the rest of the fluid bolus, generally of 350–400 mL. Over the past ten years, several studies have shown that the mini-fluid challenge reliably predicts the response to volume expansion [45].

### What's new?

#### Reliability

After relatively small studies, two recent meta-analyses confirmed the reliability of the mini-fluid challenge [45, 54]. This has been confirmed by a multicentre study involving more than 100 patients in the operating theatre [81] so that one can now reasonably consider that this is a reliable way to detect preload responsiveness (Table 1).

#### Monitoring technique

**Fig. 3:**
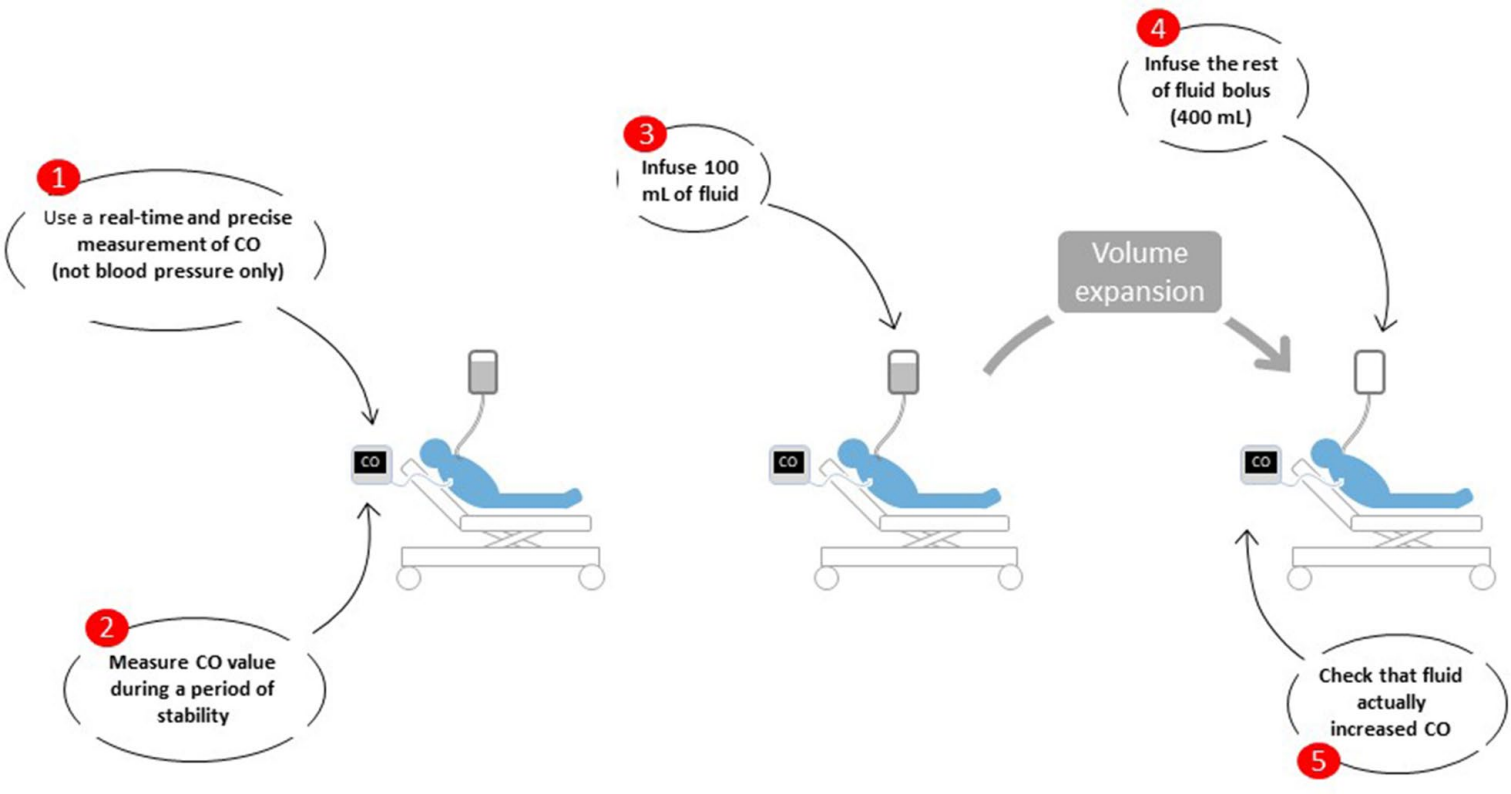
Practical rules for performing a mini-fluid challenge. *CO* cardiac output

The mini-fluid challenge requires direct estimation of cardiac output or stroke volume, that is, it cannot be done by just measuring arterial pressure changes (Fig. 3). During a classic fluid challenge, it is well demonstrated that changes in arterial pressure, even in arterial pulse pressure—best related to stroke volume—follow changes in cardiac output only very imprecisely [14, 82]. The same is obviously true for a mini-fluid challenge, the administered volume of which is smaller.

In addition, the diagnostic threshold for the mini-fluid challenge is relatively small (5% on average (Table 1) [45]). Then, the technique used for measuring cardiac output during the test must be precise, that is, it must be able to measure small changes in cardiac output (Fig. 3). The analysis of the pulse wave contour is very suitable for this, for the smallest change in cardiac output it can detect reliably is around 1–2%, as has recently been established [47]. Note, however, that even with this technique, the reliability of the mini-fluid challenge is lower if the volume infused is only 50 mL [83].

In contrast, the precision of echocardiography in measuring the LVOT VTI may not be good enough, as its least significant change is 12% [48]. So, if this is the technique that is used, one should ensure the best possible precision in estimating stroke volume. In particular, one should keep the ultrasound probe on the patient's chest during the fluid infusion, without changing the position of the ultrasound flow in the LVOT chamber, thus improving measurement precision [48].

The decrease in PPV and SVV during a mini-fluid challenge, which would indicate a decrease in the degree of preload responsiveness, might be used as a surrogate for an increase in cardiac output to assess the effects of the test. Two small studies recently showed that the decrease in PPV and SVV during a 100 mL mini-fluid challenge predicted the response to volume expansion with acceptable reliability [63, 64] (Table 1). If results obtained with PPV are confirmed, this would allow one to assess the mini-fluid challenge effects without any cardiac output monitoring, but just from an arterial pressure trace.

#### Limitations

The majority of studies that have investigated the reliability of the mini-fluid challenge pose a methodological problem [84]. In fact, these studies showed that the response to the mini-fluid challenge (100–150 mL) predicted the response to the whole fluid bolus (generally 500 mL), including that already administered during the test itself. Thus, the mini-fluid challenge predominantly predicted the effects of the mini-fluid challenge itself. This could have led to an overestimation of the test reliability [85]. Further studies should clarify whether this is a significant limitation.

Beyond this methodological limitation, the major drawback of the mini-fluid challenge is that it still involves the infusion of fluid which cannot be removed if ineffective. If repeated, it may inherently induce fluid overload, although the risk is lower than with a classic fluid challenge.

## Other tests

### Other tests using the ventilator

Recruitment manoeuvres, in the operating theatre or during acute respiratory distress syndrome, change cardiac loading conditions, and in particular, decrease cardiac preload. The decrease in stroke volume during a recruitment manoeuvre (application of continuous positive airway pressure of 30 cm H_2_O for 30 s [86, 87], or of 25 cmH_2_O for 25 s [88]) predicted the response to a subsequent bolus of fluid in surgical patients during anaesthesia [86, 88] and during one-lung ventilation [87]. Interestingly, changes in the plethysmographic perfusion index were tested under the same conditions but had less diagnostic value [89]. To our knowledge, these studies have not been repeated.

In the same vein, it was recently proposed that preload responsiveness can be tested by performing four sigh manoeuvres at 0, 15, 25 and 35 cmH_2_O and by measuring the slope of the drop in systolic blood pressure induced [90]. This test looks like the respiratory systolic variation test, which has been developed years ago [91]. It consists in measuring the slope of the lowest systolic pressure values during a standardized manoeuvre consisting of three successive incremental pressure-controlled breaths. The advantage of such tests is that they might be automated by coupling the ventilator and the haemodynamic monitoring devices.

### Trendelenburg manoeuvre

In prone positioned patients, the PLR test cannot of course be performed. However, a Trendelenburg manoeuvre, which could also transfer part of the venous blood from the lower body to the heart chambers, may be an alternative. One study showed that an increase in cardiac output during such a manoeuvre correctly detected preload responsiveness (Table 1) [56]. Note that some other tests like EEO and Vt challenge might also be suitable in the case of prone positioning. Similar results with the Trendelenburg manoeuvre have been described in patients under veno-arterial extracorporeal membrane oxygenation [92] and during the surgical intervention [93].

## Place of preload responsiveness assessment in patient management

### No magic value!

First of all, it must be remembered that no test or diagnostic index is perfect, for preload responsiveness tests or for any other tests. The further the result from the diagnostic threshold, the more likely the diagnosis. This is what some have conceptualized with the concept of a "grey zone", in which a greater or lesser number of patients are located depending on the test considered.

Additionally, it should be kept in mind that while patients are classified as "preload responders" and "preload non-responders" in studies, to determine sensitivity and specificity, the degree of preload dependence is a physiologically continuous variable. Thus, if the result of a test or index takes an intermediate value, close to the diagnostic threshold provided by the studies, this perhaps corresponds to an intermediate degree of preload dependence, not to a weakness of the diagnostic tool.

### Preload responsiveness: not always!

There are cases where the positive response of cardiac output to a bolus of fluid is certain, and in which the patient’s ventricles must work on the steep part of the Frank-Starling curve. This is certain if cardiac preload is very low. In clinical practice, this corresponds to obvious hypovolaemia, the initial phase of septic shock before any volume expansion, because of the strong relative hypovolaemia, haemorrhage… In these cases, preload responsiveness is present for sure, and testing it may only delay urgent fluid administration.

In contrast, in all other cases, fluid infusion increases cardiac output in only half of the cases, if fluid responsiveness is not tested. In this context, a dynamic measurement of preload responsiveness is recommended, as during sepsis for example [12].

### The presence of preload responsiveness does not mean that fluid should be infused

First, the assessment of fluid responsiveness cannot be dissociated from the clinical context, and no fluid should be administered if it is not obvious that cardiac output needs to be increased. There is no need to test for preload responsiveness if there is no acute circulatory failure as suggested by the absence of clinical signs of organ hypoperfusion (oliguria or anuria, increased capillary refill time, skin mottling, increase in blood lactate or veno-arterial gradient in carbon dioxide partial pressure, decrease in venous oxygen saturation). In such a case, a positive index or test of preload responsiveness must not lead to volume expansion. Preload responsiveness is a physiological state, and it makes no sense to want to fix it systematically.

Second, the need for infusing fluid boluses must be tested along with the risk of administering fluids. Even if preload responsiveness is present, if the risk exceeds the benefit, fluid should not be infused. In assessing risk, many indices may be considered, as a high central venous pressure or pulmonary artery occlusion pressure, a high level of extravascular lung water and severely impaired blood oxygenation, or an elevated intra-abdominal pressure, for instance [94]. Alternatives to fluid may be considered for improving the haemodynamic status, as using the preload effect of norepinephrine in septic shock patients [95, 96] or simply decreasing the level of PEEP, for instance.

### Testing preload responsiveness: which applications?T esting fluid responsiveness

The real need in assessing fluid responsiveness is that fluid should not be given in the case of fluid unresponsiveness, as if it is ineffective fluid will only contribute to fluid overload and its deleterious effects. It is now well established that an increase in fluid balance during an ICU stay [3] or after cardiac surgery [4] worsens patient outcome, regardless of other factors of severity.

From this perspective, testing preload responsiveness can also serve to guide fluid removal at the therapeutic de-escalation phase in shock patients. Indeed, in the absence of preload responsiveness, it is likely that the fluid can be withdrawn in a safe manner, without the risk of lowering cardiac output and causing hypotension [9]. In addition, an absence of preload responsiveness, which indicates the inability of the heart to cope with significant changes in its loading conditions, makes it likely that weaning from mechanical ventilation will fail due to cardiac dysfunction [97].

### Testing fluid responsiveness… and assessing the response to fluids

If the detection of a preload responsive state is to precede the administration of fluid, it is important to verify, if a bolus of fluid has been administered, that it has indeed increased cardiac output (Figs. 1, 2, 3). First, none of the diagnostic methods are perfect, and false positives or false negatives are always possible. Then, if a significant response of cardiac output to fluid administration is noted, it is possible that a preload responsiveness state persists, so that the question of renewing a fluid bolus may arise.

Finally, the ultimate goal of volume expansion is to correct tissue hypoxia. Due to the non-linear relationship between oxygen consumption and oxygen delivery in some cases, the fluid-induced increase in cardiac output is not always accompanied by improved tissue oxygenation [98]. It is therefore important to evaluate it according to the usual indices (skin mottling, capillary refill time, lactate, venous oxygen saturation and carbon dioxide-derived indices) [94].

### Any effect on patient outcome?

Recent studies and meta-analyses have addressed the issue of whether a strategy guided by the assessment of preload responsiveness improves patient outcome, in the ICU or in the operating theatre.

It seems that these two settings should be considered differently. In the operating theatre, there are quite a few studies comparing an interventional fluid strategy guided by preload responsiveness assessment with a standard strategy. They were of relatively small size, but their meta-analyses suggested a benefit of the interventional fluid strategy regarding different outcomes. In a 2017 meta-analysis including 13 trials (1652 patients), 12 of which were performed in post-surgical patients, a fluid strategy based on a fluid responsiveness assessment significantly decreased mortality and ICU length of stay [99]. This meta-analysis confirmed the results of an earlier one, performed specifically in post-surgical patients, showing that a goal-directed strategy based on dynamic parameters decreased post-surgical morbidity and ICU length of stay [100]. This was confirmed in a meta-analysis of 11 studies (1015 patients) performed in surgical ICU patients and in which a strategy based on SVV reduced the ICU and hospital lengths of stay and tended to decrease mortality [101].

In addition, in the peri-operative period, several studies have examined the effect on outcome of using a goal-directed therapy, which included, in addition to other therapeutic interventions, a fluid strategy guided by assessing preload responsiveness. Several of these studies and their meta-analyses [102] have shown that such a goal-directed approach is beneficial, especially in decreasing post-operative complications. A recent meta-analysis including 21 randomized control trials enrolling 2729 patients found that goal-directed therapy was associated with a reduction in post-operative complications and a trend toward reduced mortality [103].

In non-surgical ICU patients, far fewer studies are available. Three randomized control trials only have been published in English in peer-reviewed journals [104–106] (Table 3). They were all performed on septic shock patients in the early phase, and all used the PLR test to assess preload responsiveness in the protocol group. As summarized in Table 3, in two of them, the primary goal of reducing the volume of fluid administered through a PLR-guided strategy was achieved. In addition, the trial that included the largest number of patients evidenced a reduction in the need for renal replacement therapy and for mechanical ventilation in the intervention group [104]. A reduction in mortality, which was not the primary goal of these studies, was not observed, as confirmed in two meta-analyses [107, 108].

**Table 3:** Summary of the studies investigating the effects on the outcome of strategies using an assessment of preload responsiveness of critically ill patients

First author (year of publication)	Number of patients	Number of centres	Primary end-point	Effect of fluid administration*		Effect on mortality*		Other tested effects*	
Chen [96]	82	1	Volume of fluids administered by days 3 and 5 and cumulative fluid balance by days 3 and 5	Fluid balance at Day-3	1 952 [48–5003] mL vs 3 124 [767–10103] mL, *p* = 0.20	In-hospital	56% vs. 49%, *p* = 0.51	Ventilator-free days	5.5 [0–12.25] days vs 5.5 [0–16.75] days, *p* =0 .05
								Need for renal replacement therapy	41.5% vs 39.0%, *p* = 0.82
								Vasopressor-free days	5.5 [0–10] days vs 5 [0–16] days, *p* = 0.84
Richard [97]	60	1	Duration of cardiovascular failure	Daily volume of fluids for volume expansion	383 (211 to 604) mL/day vs 917 (639 to 1,511) mL/day, ***p* = 0.01**	28-day	23% vs. 47%, *p* = 0.10	Time to shock resolution	2.0 (1.2 to 3.1) days vs 2.3 (1.4 to 5.6) days, *p* = 0.29
								Red cell transfusions	103 (0 to 183) mL vs 178 (82 to 304) mL, ***p* = 0.04**
								Ventilator-free days	14 [0–24] days vs 8 [0–21] days, p = 0.35
Douglas [95]	150	13	Positive fluid balance at 72 h or ICU discharge	Fluid balance at 72 h or ICU discharge	0.65 ± 2.85 L vs 2.02 ± 3.44 L, ***p* = 0.02**	30-day	20% vs. 21%, *p* = 0.42	Need for rate of renal replacement therapy	5.1% vs 17.5%, ***p* = 0.04**
								Need mechanical ventilation	17.7% ± 34.1%, ***p* = 0.04**

Results in the intervention arm are presented first, and results in the control arm second. P values <0.05 are indicated in bold.

*ICU* intensive care unit

Finally, in a multicentre observational study in Argentina including 787 septic shock patients, the use of various indices and tests assessing fluid responsiveness was associated with a better outcome in logistic regression analysis [109]. Nevertheless, the observational nature of the study precludes any definitive conclusion.

At the very least, it can be said that a strategy using the assessment of preload responsiveness in septic shock patients is certainly not deleterious and that it does not excessively delay therapeutic management.

However, even if further studies on larger numbers of patients were carried out, it is not at all certain that they could show improved survival. Indeed, a decrease in mortality may not be the ideal outcome for demonstrating the benefit of a treatment in critically ill patients [110]. In patients as heterogeneous as those with circulatory failure, in a disease as multifactorial as shock, it is not certain that any reasonable management algorithm can be established, nor that the change of only one aspect of treatment can demonstrate an effect on survival. In addition, demonstrating the effect of any intervention on outcome requires a large difference between the intervention and the control groups. Such a demonstration may be more difficult today than earlier, as the outcome of the control group has improved in many instances.

Finally, should we wait for the results of survival studies to implement methods assessing preload responsiveness in clinical practice? Since these methods are harmless, as they often use cardiac output monitoring which is recommended anyway in shock patients [111] and patients at high surgical risk [102], they could be chosen for the simple reason that they allow potentially dangerous treatment to be administered only to patients who benefit from it. It seems logical to condition the infusion of fluid boluses on the presence of preload responsiveness, simply because it protects patients from the useless administration of dangerous drugs. This can only help tailor the treatments, as we should strive for, especially during septic shock [112].

## Conclusion

The most recent studies in the prediction of fluid responsiveness have primarily described means of measuring the effects of well-established preload responsiveness tests, such as the PLR test, the EEO test and the mini-fluid challenge. In particular, methods replacing invasive and costly measurements of cardiac output have been described. In addition, the limits of these tests have been better defined. Some recent studies have also developed and validated new tests, the best validated of which is the Vt challenge, which has the advantage of being assessed with no direct estimation of cardiac output. There is now some evidence that in the peri-operative period the use of a therapeutic strategy adapting fluid resuscitation to the detection of preload responsiveness reduces post-operative complications. In non-surgical critically ill patients, such as septic shock patients, few outcome studies have been performed, suggesting a reduction in the amount of administered fluid.

## Abbreviations

EEO: End-expiratory occlusion
ICU: Intensive care unit
LVOT: Left ventricular outflow tract
PLR: Passive leg raising
PPV: Pulse pressure variation
SVV: Stroke volume variation
Vt: Tidal volume
VTI: Velocity time integral
